# Is There Less Alteration of Smell Sensation in Patients With Omicron SARS-CoV-2 Variant Infection?

**DOI:** 10.3389/fmed.2022.852998

**Published:** 2022-04-12

**Authors:** Juan Jose Rodriguez-Sevilla, Roberto Güerri-Fernádez, Bernat Bertran Recasens

**Affiliations:** ^1^Hematology Department, Hospital del Mar, Barcelona, Spain; ^2^Infectious Diseases Department, Hospital del Mar Institute of Medical Research (IMIM), Barcelona, Spain; ^3^Facultad de Medicina y Ciencias de la Vida (MELIS), Universitat Pompeu Fabra, Barcelona, Spain; ^4^Neuromuscular Unit, Neurology Department, Hospital del Mar, Barcelona, Spain

**Keywords:** COVID-19, anosmia, Omicron variant, inflammation, vaccines

## Abstract

The ongoing pandemic Coronavirus Disease 2019 (COVID-19) caused by the severe acute respiratory syndrome coronavirus 2 (SARS-CoV-2) is a matter of global concern in terms of public health Within the symptoms secondary to SARS-CoV-2 infection, hyposmia and anosmia have emerged as characteristic symptoms during the onset of the pandemic. Although many researchers have investigated the etiopathogenesis of this phenomenon, the main cause is not clear. The appearance of the new variant of concern Omicron has meant a breakthrough in the chronology of this pandemic, presenting greater transmissibility and less severity, according to the first reports. We have been impressed by the decrease in anosmia reported with this new variant and in patients reinfected or who had received vaccination before becoming infected. Based on the literature published to date, this review proposes different hypotheses to explain this possible lesser affectation of smell. On the one hand, modifications in the SARS-CoV-2 spike protein could produce changes in cell tropism and interaction with proteins that promote virus uptake (ACE-2, TMPRSS2, and TMEM16F). These proteins can be found in the sustentacular cells and glandular cells of the olfactory epithelium. Second, due to the characteristics of the virus or previous immunity (infection or vaccination), there could be less systemic or local inflammation that would generate less cell damage in the olfactory epithelium and/or in the central nervous system.

## Introduction

Neurological symptoms such as loss of taste and, specifically, olfactory dysfunction have been consistently reported within the different SARS-CoV-2 variants (1, 2). Olfactory dysfunction, defined as the reduction (hyposmia) or total loss (anosmia) of smell during sniffing or eating, better predicts the disease than other well-known symptoms such as fever and cough (3, 4). Despite the high incidence of these symptoms, underlying mechanisms have been unclear (5). Hyposmia was not initially recognized to be linked to COVID-19 and was only mentioned to affect about 5% of COVID-19 patients in one of the first studies from China (1). However, a much higher prevalence was reported in subsequent studies from Europe, the Middle East, and North America (6–9). A recent systematic review and meta-analysis with 23,353 patients diagnosed with COVID-19 (10) pointed out that prevalence of smell loss and taste loss were 38 and 30.35%, respectively, with significant differences between Western countries and East Asian ones. Although there is a great plethora of factors related to the appearance of this phenomenon, we must not forget the important advances that are being made with regard to host genetic factors (11) and their implication in susceptibility to infection, immune response, and even symptomatology accompanying SARS-CoV-2 infection.

One of the possible rationales could implicate that Caucasians have more often an Angiotensin-converting enzyme 2 (ACE2) variant expressed in the olfactory epithelium (12, 13) (presumably in the sustentacular cells of the olfactory epithelium). Because this protein is one of those used by the receptor binding domain (RBD) of the SARS-CoV-2 Spike Protein to infect the cell (14), genetic variability and the mutation rate within the RBD domain is of particular interest in the context of population differences in the prevalence of hyposmia.

Recently, the Norwegian Institute of Public Health reported the demographics of 117 cases infected with Omicron variant (15). Strikingly, loss of smell and taste was reported in 12% (median duration 2 days) and 23% (median duration 2.5 days), respectively (15). This feature has also been supported by US State Department of Health (16) and by others studies (17, 18).

Vihta et al. (17) showed loss of smell and taste was found to be less common among Omicron compared to Delta cases (13% of Omicron cases, 34% of Delta cases, odds ratio 0.22, 95% CI: 0.21–0.23). Moreover, Boscolo-Rizzo et al. (18), in a pre-printed version, presented data on a prospective study on mild-to-moderate symptomatic adult patients and reported that 24.6% of patients had smell alterations during the proxy Omicron period compared to 62.6% during the comparator period mainly driven by the delta variant (*p* < 0.001). Finally, a recent report led by Maisa et al. collecting 468 Omicron cases in different regions of France reported an 8.3% of anosmia (19).

The Omicron (B.1.1.529) COVID-19 variation of concern (VOC) was first identified in South Africa on November 9, 2021 (20). This variant was accounted for to the World Health Organization (WHO) by South African experts on November 24, 2021, after which the WHO's Technical Advisory Group on SARS-CoV-2 Virus Evolution (TAG-VE) was reconvened on November 26, 2021, which prompted B.1.1.529 to being meant as a VOC (21).

The omicron variant seems to be highly transmissible, presenting many substitutions in the spike glycoprotein and appeared at a time when a large part of the world population had received SARS-CoV-2 vaccination.

**Figure 1:**
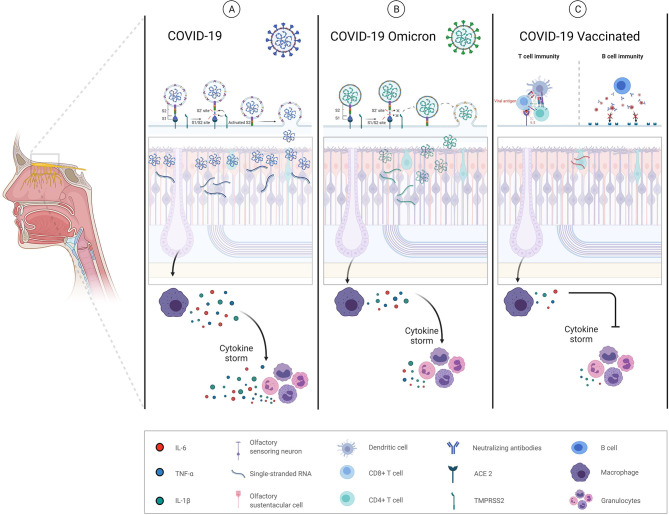
Schematic mechanism proposal of anosmia/hyposmia in COVID-19. **(A)** SARS-CoV-2 alpha variant. (1) Infection and destruction of olfactory epithelial supporting cells (olfactory sustentacular cells), resulting in inflammation, and abnormalities in local homeostasis; (2) infection or immune-mediated damage of surrounding cells (vascular cells) resulting in hypoperfusion, inflammatory cell recruitment, cytokine release, and the production of chemical neurotoxins. **(B)** SARS-CoV-2 Omicron variant. Omicron mechanism of cell entry based on a TMPRS22 dependent and non-dependent manner may involve a more heterogenous tropism with less replication and the consequent minor local inflammation produced. **(C)** SARS-CoV-2 vaccines may not alter the levels of replicating virus in the olfactory epithelium once viral infection establishes but may instead protect through accelerated clearance or enhanced neutralization of infectious viral particles.

We hypothesize that Omicron variant features or previously acquired immunity, either due to previous infection or vaccination, could explain the lower incidence of olfactory disturbances. Omicron could produce less hyposmia due to differences in cell tropism, mechanism of entry into cells and by producing less inflammatory dysregulation (Figure 1).

## Mechanisms of Hyposmia

Due to its significant prevalence, hyposmia has been one of the neurological symptoms that has aroused the greatest interest in the scientific community. A wide-variety of scenarios such as olfactory cleft syndrome (22), systemic and local inflammation in the olfactory epithelium, apoptosis of the olfactory receptor neurons and sustentacular cells, changes in the olfactory cilia and odor transmission, injury of microglial cells, effect on the olfactory bulb, epithelial olfactory injury (e.g., Sustentacular cells), as well as damage of olfactory receptor neurons and olfactory stem cells neurons have been described as possible mechanisms (23). Damage to these olfactory stem cell neurons may be the cause for persistent anosmia after COVID-19 recovery despite having acquired specific immunity.

## Hyposmia in Omicron SARS-CoV-2 Variant

### Tropism and Entry Mechanism

The omicron variant seems to be highly transmissible, presenting many substitutions in the spike glycoprotein. Several mutations in the RBD and S2 region of the spike protein are predicted to impact transmissibility and affinity for the ACE-2 receptor (24). Brann et al. found that the gene encoding the ACE2 receptor protein, which SARS-CoV-2 utilizes to enter human cells, is not expressed in olfactory sensory neurons (OSNs) (25). Nevertheless, two specific cell types in the olfactory epithelium expressed ACE2 at similar levels to what has been observed in the lower respiratory tract cells. These included sustentacular cells, which embraced sensory neurons and are thought to provide structural and metabolic support, and basal cells, which act as stem cells that regenerate the olfactory epithelium after damage. ACE2 receptors are also found in glial cells, neurons and capillary endothelium and the interaction with the virus could be one of the mechanisms that allows it to enter into the central nervous system (26).

The key to understand such differences in infectivity of SARS-CoV-2 may lie in the frequency of variants in the virus entry proteins, ACE2, and Transmembrane serine protease 2 (TMPRSS2), which may depend on cell type and racial or ethnic groups (27, 28).

Recently, Bentley et al. studied Omicron features using an established K18-hACE2 mouse model of SARS-CoV-2 (29). The infection of hACE2 mice with Omicron variant led to less viral load in oral swabs and nasal tissue and to less severe clinical signs and less severe pneumonia compared with Delta variant. However, more studies are needed because McMahan et al. suggested that Omicron infection may lead to increased upper respiratory tract disease but reduced lower respiratory tract disease (30) compared with WA1/2020 variant. These results should be taken cautiously since no animal model can predict with absolute certainty the consequences of infection in humans. Meanwhile, Hong Kong investigators studied Alpha, Beta, Delta and Omicron variants in *ex vivo* explant cultures of human bronchus and lung. They suggested that B.1.1.529 replicates faster in the human bronchus and less in lung cells, which may explain its greater transmissibility and putative lower disease severity (31). At the same time, they propose a less-dependent TMPRSS2 activation in Omicron variant compared with Delta, suggesting that Omicron may have a broader spectrum of target cells compared to Delta (32). This last proposal was also supported by Thomas et al., showing that Omicron achieved this rapid replication rate by becoming less specialized in its cellular tropism, entering cells in both a TMPRSS2-dependent and –independent manner (33). Recently it was proposed by Meng et al. that TMPRSS2 usage may be impacted by ACE2 levels after studies in organoid systems and human nasal epithelial cultures (34).

While the switch in passage pathway has expanded its intrinsic contagiousness, the less effective utilization of cell surface TMPRSS2 by Omicron Spike protein has also brought about a diminishing affinity for syncytia arrangement (34). This may explain the decreased disease severity impression since syncytia have been reported in the autopsy of COVID cases and the efficient cleavage at the furin site that underlies syncytia formation has been associated with enhanced disease severity in animal models (35).

Lastly, another essential aspect that should be considered is the fusogenic capacity of this new SARS-CoV-2 VOC. Recently, Meng et al. showed Omicron spike is relatively poorly cleaved and impaired in mediating cell-cell fusion and syncytia formation (34). This observation is of interest because, for syncytia formation, Transmembrane 16F (TMEM16F) protein is one of the proteins that are activated by the SARS-COV-2 spike (35). This calcium-activated chloride channel is also located on the cilia of olfactory sensory neurons and appears to be involved in olfactory signal transduction (35, 36). Perhaps Omicron variant does not alter these proteins, and this could explain, in part, the lower incidence of olfactory alterations.

In summary, the different data from *ex vivo*, mouse model or *in vitro* studies suggest that the Omicron variant has a particular cellular entry mechanism that may explain the lower incidence of olfactory impairment. Still such interpretations need to be qualified because the semiotics of COVID-19 is determined not only by virus replication but also by dysregulated innate immune responses.

### Inflammation

Olfaction is a complex process comprising multiple components, including receptors, nerves, and structures of the brain. Cell-signaling processes are critical in any complex sensory system such as olfaction, where a myriad selection of cytokines (37) or even intermediate metabolic substances such as zinc play an essential role (38).

Before the COVID-19 breakthrough, IL-6 was already considered a possible causal factor for initiation of hyposmia reflective of local or systemic immunological/inflammatory changes in blood, saliva, or nasal mucus (39). This hypothesis is consistent with finding smell loss among patients with inflammatory rheumatoid arthritis (40). Elevated IL-6 level has been previously reported in nasal lavage fluid from patients with naturally acquired parvovirus (41), in addition, other viruses have been found in turbinate epithelial cells of patients with post-viral olfactory dysfunction (42).

Sensorineural olfactory loss could occur due to destruction of the olfactory neuroepithelium by toxic inflammatory factors (such as TNF-α, IL-6, and IL-1β) (43). Cazzolla et al. observed in 67 COVID patients that the resolution of olfactory alterations is accompanied by a progressive decrease of IL-6 levels to normal values (44). Thus, a new approach is opened in which the decrease of IL-6 from the beginning to the disappearance of the symptoms would have greater clinical relevance than the initial peak values of IL-6. Elevated IL-6 level could act as an endogenous substance regulating olfactory neuronal activity because it has been shown to regulate neuronal (45) and glial cell activity (46). For instance, Neuropoietin, an IL-6–related cytokine that affects signaling through ciliary neurotrophic factor receptor (47), could directly inhibit smell function because inhibition of several ciliary factors has induced smell loss in patients with other syndromic disorders (48, 49).

In contrast to these findings, Bax et al. failed to associate different inflammatory markers (CRP, IL-6) with the presence or absence of anosmia in 93 COVID patients (50). However, it is possible that any nasal IL-6 production within the olfactory epithelium is insufficient to contribute significantly to serum IL-6 levels.

Finally, Torabi et al. analyzed TNF-α and IL-1β levels in olfactory epithelial biopsies from patients with confirmed COVID-19 and uninfected controls (51). In this study, the authors proposed that the inflammatory infiltration that occurs in response to local TNF-α expression may lead to a considerable expansion of the olfactory submucosa and causes damage to OSN (52).

A plausible scenario would be one in which the Omicron variant would not markedly produce such a pro-inflammatory environment in the olfactory epithelium and/or central nervous system, providing additional explanation for the low rate of anosmia and hyposmia reported with this variant.

### Role of Acquired Immunity

About 50 mutations have been detected in the Omicron variant (30 in the spike protein) (20). These mutations, especially those of the spike protein, make the virus capable of evading, at least in part, the neutralizing antibodies generated by vaccination or previous infections.

However, beyond generating a specific humoral response, both vaccination and previous infections produce a longer-lasting cellular response mediated by CD4 and CD8 T-cells. This response seems to be maintained at 70–80% against the Omicron variant (53–55).

In addition to the intrinsic characteristics of the Omicron variant, this previous specific immunity could explain why the new variant generates less severe infection and causes a lesser and less lasting cytokine storm.

The fact of generating a lower inflammatory response in the olfactory epithelium and a faster elimination of the virus could explain the lower incidence of hyposmia. It has already been shown that vaccinated patients (especially with two doses), who are re-infected by SARS-CoV-2 (before the appearance of the Omicron variant) had a lower incidence of smell alterations and duration of symptoms was shorter (56, 57). However, the only data we have found from the smell loss registry in symptomatic COVID-19 patients after fully vaccination suggest that anosmia/hyposmia may be an existing finding in these subjects (58). Further studies involving close and personalized symptom monitoring together with a correct genome characterization of SARS-CoV-2 are necessary to shed light on this topic.

## Where Next?

Since the advent of the Omicron variant, the general impression in the medical community is that there is a lower incidence of hyposmia in infected people. Despite the lack of studies with large sample size, preliminary data from Norway have reported only 12% alterations in smell compared to 38% in the other variants (15).

Among the causes that may explain this lower incidence are the characteristics of the new variant and its interaction with the organism and its response to infection, and, finally, the role of previously acquired immunity (by previous infection or vaccination).

The Omicron variant presents a series of mutations in the spike protein that affect the affinity for the ACE2 receptor, generate a less specialized cell tropism (cell entry based on a TMPRS22 dependent and non-dependent manner) and, finally, a lower capacity for cell fusion. On the other hand, because the new variant appears less pathogenic, it may generate a lower viral load and less local or systemic inflammation (e.g., IL-6, TNF-α, IL-1β, etc.).

Finally, one of the most plausible explanations is that the previous immunity generated by vaccines or past infections causes less local or systemic inflammation for less time and faster viral elimination.

However, we must not ignore that the different possible explanations that we propose in this manuscript should be taken cautiously. Several elucidations have been suggested (55); nevertheless; we are aware that some of them may not be verified in the future and others are based on unreviewed preprinted articles which will be needing future validation. In order to delve into this phenomenon, more studies are required to establish the exact mechanisms of how SARS-CoV-2 elicits olfactory system damage.

In conclusion, less direct damage by the virus and less inflammation—either due to virus characteristics or previous immunity—could explain less damage to the olfactory epithelium and/or central nervous system. Larger sample size studies are needed to establish the incidence of olfactory alterations in patients with Omicron, to be able to associate them with certain risk factors and to better understand the etiopathogenesis.

## Data Availability Statement

The original contributions presented in the study are included in the article/supplementary material, further inquiries can be directed to the corresponding author/s.

## Author Contributions

JR-S: study concept and design and figure editing. JR-S and BB: methodology, literature review, data collection, analysis, interpretation, writing-original draft manuscript, and review-original final manuscript. All authors have read and agreed to the published version of the manuscript.

## Funding

This work has received support from FEDER funds and the FIS project form Instituto de Carlos III (PI19/00019).

## Conflict of Interest

The authors declare that the research was conducted in the absence of any commercial or financial relationships that could be construed as a potential conflict of interest.

## Publisher's Note

All claims expressed in this article are solely those of the authors and do not necessarily represent those of their affiliated organizations, or those of the publisher, the editors and the reviewers. Any product that may be evaluated in this article, or claim that may be made by its manufacturer, is not guaranteed or endorsed by the publisher.
